# Regulatory Pathways of Monoamine Oxidase A during Social Stress

**DOI:** 10.3389/fnins.2017.00604

**Published:** 2017-10-31

**Authors:** Yuki Higuchi, Tomoko Soga, Ishwar S. Parhar

**Affiliations:** Brain Research Institute, School of Medicine and Health Sciences, Monash University Malaysia, Bandar Sunway, Malaysia

**Keywords:** serotonin, HPA axis, depression, monoamine oxidase, 5-hydroxyindoleacetic acid

## Abstract

Social stress has a high impact on many biological systems in the brain, including serotonergic (5-HT) system—a major drug target in the current treatment for depression. Hyperactivity of hypothalamic-pituitary-adrenal (HPA) axis and monoamine oxidase A (MAO-A) are well-known stress responses, which are involved in the central 5-HT system. Although, many MAO-A inhibitors have been developed and used in the therapeutics of depression, effective management of depression by modulating the activity of MAO-A has not been achieved. Identifying the molecular pathways that regulate the activity of MAO-A in the brain is crucial for developing new drug targets for precise control of MAO-A activity. Over the last few decades, several regulatory pathways of MAO-A consisting of Kruppel like factor 11 (KLF11), Sirtuin1, Ring finger protein in neural stem cells (RINES), and Cell division cycle associated 7-like protein (R1) have been identified, and the influence of social stress on these regulatory factors evaluated. This review explores various aspects of these pathways to expand our understanding of the roles of the HPA axis and MAO-A regulatory pathways during social stress. The first part of this review introduces some components of the HPA axis, explains how stress affects them and how they interact with the 5-HT system in the brain. The second part summarizes the novel regulatory pathways of MAO-A, which have high potential as novel therapeutic targets for depression.

## Introduction

Depression is one of the most prevalent mental disorders (Kessler and Bromet, 2013). In Diagnostic and Statistical Manual of Mental Disorders V, depressive disorders include major depressive disorder (MDD), premenstrual dysphoric disorder, persistent depressive disorder (dysthymia), disruptive mood dysregulation disorder, substance/medication-induced depressive disorder, and unspecified depressive disorder. Although, these types of depressive disorders are differentiated by the duration, timing, and causes of the symptoms, they have common features, such as the presence of sad, empty or irritable mood, occurring with somatic and cognitive changes. Although, many therapeutic drugs have been released in the market and used for treatment, clinicians still encounter many difficulties in the diagnosis and therapeutics of depressive disorders. It has been thought that depressive symptoms are induced by multiple factors, such as stressful life events, genetic factors, substance use, and personality (American Psychiatric Association, 2013).

Stress is one of the primary risk factors contributing to the development of many psychiatric disorders, including depressive disorders, post-traumatic stress disorder, and anxiety disorders. Definitions of stress and stressor are often confused with each other in stress research. Hans Selye, a pioneer in stress research, who coined the term “stress” in biology and medicine, defined stress as “the nonspecific response of the body to any demand” and a stressor as “an agent that produces stress at any time” (Selye, 1976). However, nowadays the word stress is sometimes used to refer to the factors that cause the response of the organisms. In stress research, the influence of stress on the biological system has been evaluated by using the following stress in different categories; (1) physical stress (e.g., mechanical trauma and infection), (2) environmental stress (e.g., inappropriate humidity and temperature), (3) psychological stress (e.g., social isolation, social defeat, maternal separation, and unpredictable environment). Among many types of stress, social stress is often recognized as one of the common causes of depression. Social stress is defined as “a situation which threatens one's relationships, esteem, or sense of belonging within a dyad, group, or larger social context” (Juth and Dickerson, 2013). In humans, stress mainly stems from relationships with others, which may happen throughout the life. Bullying in schools and harassment in workplaces are the typical examples of social stress. Statistically, depressive patients experience stressful life events at a significantly higher percentage, compared to psychiatrically normal subjects (Mazure, 1998; Kendler et al., 1999).

The hypothalamic-pituitary-adrenal (HPA) axis is a well-documented endocrine system that regulates the biological response to various types of stress, including social stress (Walker et al., 2009; Backström and Winberg, 2013), maternal separation (Aisa et al., 2008; Nishi et al., 2014), and restraint stress (Ver Hoeve et al., 2013). In brief, stress perceived by animals promotes the release of corticotropin-releasing hormone [CRH; also referred to as corticotropin releasing factor (CRF)] from the hypothalamus, which facilitates the secretion of adrenocorticotropic hormone (ACTH) from the pituitary gland to the systemic circulation (Rivier and Plotsky, 1986). Subsequently, glucocorticoids, such as cortisol and corticosterone, are released from the adrenal cortex, following the increase of ACTH levels in the blood (Papadimitriou and Priftis, 2009). Although, elevated cortisol levels are observed in depressive patients (Sachar et al., 1970; O'Brien et al., 2004), it is not a common characteristic feature of depression among all patients (Strickland et al., 2002). There are differences shown in the pattern of cortisol secretion, depending on subtypes of depression. A meta-analysis on the association between depression and cortisol levels indicates that cortisol response to psychosocial stress depends on age and symptom severity of depressive patients (Burke et al., 2005). Further, it has been shown that recurrent subtype of depression is associated with higher cortisol levels (Peeters et al., 2004). A clinical study divided the depressed patients into hypercortisolemic group and non-hypercortisolemic group, and found that melancholic and psychotic depressive subtypes are strongly associated with higher cortisol levels in depressive patients (Carroll et al., 2007).

## Hypothalamus-pituitary-adrenal axis during stress

### CRH system in social stress

CRH is a neuropeptide which consists of 41 amino acids in human, rodents, and fish (Vale et al., 1981; van Enckevort et al., 2000). Human CRH is synthesized from the preproCRH peptide of 196 amino acids (Robinson et al., 1989). Amino acid sequences of mature CRH in human, rat, and mouse are fully identical (Reviewed in Majzoub, 2006). A high CRH density is observed in the parvocellular neurons in the paraventricular nucleus of the hypothalamus, where the majority of this peptide is synthesized (Bloom et al., 1982; Raadsheer et al., 1993).

CRH is not the only mediator between the stress stimuli and ACTH secretion from the pituitary gland but a neuronal factor, which interacts with the brain 5-HT system during stress response. CRH terminals contacting 5-HT-containing dendrites are observed in the dorsal raphe nucleus (Waselus et al., 2005). Furthermore, the serotonergic dorsal raphe nucleus expresses CRH receptors (Chalmers et al., 1995; Wood et al., 2013). Intracerebroventricular and intraraphe administrations of CRH inhibits the activity of serotonergic neurons in the dorsal raphe nucleus in rats (Price et al., 1998). Direct administration of CRH into the dorsal raphe nucleus decreases the extracellular 5-HT levels in the terminal regions (the striatum and the lateral septum), implying that CRH may regulate the 5-HT release from serotonergic neurons via dorsal raphe nucleus (Price and Lucki, 2001). Interestingly, CRH has biphasic effects on 5-HT levels in the brain. Intracerebroventricular administration of CRH at lower dose decreases the extracellular 5-HT levels in the striatum and nucleus accumbens, whereas at higher dose CRH increases the extracellular 5-HT levels in these brain regions (Price et al., 1998; Lukkes et al., 2008).

5-HT signals influence the HPA-axis through the regulation of CRH levels in the brain. Intracerebroventricular injection of 5-HT induces the increase in CRH gene expression in the hypothalamic paraventricular nucleus of rats, which is abolished by pretreatment with a selective 5-HT_1A_ receptor antagonist or a selective 5-HT_3_ receptor antagonist, rather than a selective 5-HT_2A/2C_ receptor antagonist (Kageyama et al., 1998). These results suggest that CRH release is mediated by 5-HT_1A_ and 5-HT_3_ receptors. On the contrary, CRH mRNA expression is also increased by the intracerebroventricular administration of 5-HTP (precursor of 5-HT) and fluoxetine (selective serotonin reuptake inhibitor) as well as the intracerebroventricular administration of specific 5-HT_1A_, 5-HT_1B_, 5-HT_2A_, or 5-HT_2C_ receptor agonist but not by specific 5-HT_3_ receptor agonist (Jørgensen et al., 2002).

The effect of CRH is mediated by two different receptors, corticotropin releasing hormone receptor 1 (CRH-R1) and corticotropin releasing hormone receptor 2 (CRH-R2). Both of them are categorized as G-protein coupled receptors. CRH-R1 has an N-terminal extracellular domain, which is crucial for high-affinity ligand binding (Dautzenberg et al., 1998; Wille et al., 2002). CRH-R2 has an N-terminal structure different from that of CRH-R1 and different ligand binding property (Grigoriadis et al., 2001). In rodents, CRH-R1 mRNA is predominantly expressed in the cerebral cortex, sensory relay nuclei and cerebellum in the brain and the intermediate and anterior lobe of the pituitary gland (Potter et al., 1994; Van Pett et al., 2000). Major sites of CRH-R2 mRNA expression in rat and mouse brains includes the olfactory bulbs, the lateral septal nucleus, the bed nucleus of the stria terminalis, the ventromedial nucleus of the hypothalamus, the medial and posterior cortical nuclei of the amygdala, the interpeduncular nuclei, mesencephalic raphe, the non-neuronal elements of the choroid plexus (Chalmers et al., 1995; Van Pett et al., 2000), and the nucleus of solitary tract (Van Pett et al., 2000). In the pituitary gland, CRH-R2 mRNA is mainly localized in the posterior lobe of the pituitary gland of rats and mice (Van Pett et al., 2000).

Social defeat increases CRH-R1 mRNA levels in the prefrontal cortex (Boutros et al., 2016), cingulate, prelimbic, and in the infralimbic (Uribe-Mari-o et al., 2016). Social stress promotes the internalization of CRH-R1 in 5-HT neurons in the dorsal raphe (Wood et al., 2013). Social defeat stress activates the neuronal activity as measured by c-Fos-positive cell numbers in the medial amygdala where CRH-R2 mRNA is co-localized (Fekete et al., 2009). The existence of two subtypes of CRH receptors in 5-HT neurons explains the biphasic effects of CRH on the extracellular 5-HT levels in the brain. In the study conducted by Lukkes and co-workers, a decrease in the accumbal 5-HT levels, caused by low-dose CRH administration was abolished by antagonizing the CRH-R1 in the dorsal raphe nucleus, while an increase in the accumbal 5-HT levels induced by higher dose CRH administration was blocked by antagonizing the CRH-R2 in the dorsal raphe nucleus (Lukkes et al., 2008). This result implies that the CRH in the dorsal raphe nucleus might activate the two subtypes of CRH receptors differently depending on their concentration in the dorsal raphe nucleus.

In addition to these two types of receptors, there is a protein called CRH-binding protein (CRH-BP), which binds CRH with high affinity and thus might reduce the effects of CRH (Potter and Vale, 1991; Woods et al., 1994; Cortright et al., 1995; Seasholtz et al., 2002). CRH-BP is predominantly expressed in the cerebral cortex, although there are some prominent sites of expression in the amygdala, the bed nucleus of the stria terminalis, the dorsomedial and ventral premammillary nuclei and several raphe nuclei (Potter et al., 1992). Some studies have indicated the role of CRH-BP in non-social stress conditions, such as restraint stress (McClennen et al., 1998; Lombardo et al., 2001; Herringa et al., 2004) and food deprivation (Timofeeva et al., 1999). In rodents, studies related to the relationship between social stress and CRH-BP in the brain are largely lacking. However, there is only one study that showed the lack of effect of social stress on CRH-BP mRNA levels in the hippocampus (Marini et al., 2006). Changes in the CRH system induced by social stress are summarized in Table 1.

**Table 1:** Factors changed by social stress in animal models or depression in human.

**Factors**	**Changes**	**Species**	**References**
CRH	↑	Mouse	Keeney et al., 2006
	↓	Rat	Boutros et al., 2016
	↓	*A. burtoni*	Chen and Fernald, 2008
CRH-R1	↑	Rat	Boutros et al., 2016
	↓	*A. burtoni*	Chen and Fernald, 2008
CRH-BP	↑	Rainbow trout	Alderman et al., 2008
	↑	*A. burtoni*	Chen and Fernald, 2008
5-HT	↑	Mouse	Keeney et al., 2006
	↓	Rat	Grunewald et al., 2012
5-HIAA/5-HT ratios	↑	Rainbow trout	Winberg and Lepage, 1998; Overli et al., 1999
	↑	Mouse	Beitia et al., 2005
5-HT_1A_ receptors	↓	Mouse	Boyarskikh et al., 2013
	↑	Human	Miller et al., 2009a; Parsey et al., 2010
	↓	Human	López-Figueroa et al., 2004
5-HT_1B_ receptors	↑	Human	Belzeaux et al., 2010
	↓	Human	Murrough et al., 2011
SERT	↓	Mouse	Boyarskikh et al., 2013
	↓	Human	Miller et al., 2009b; Reimold et al., 2011; Kambeitz and Howes, 2015
MAO-A	↑	Rat	Grunewald et al., 2012
	↓	Mouse	Boyarskikh et al., 2013
	↑	Human	Meyer et al., 2006, 2009; Johnson et al., 2011; Sacher et al., 2015
KLF11	↑	Rat	Grunewald et al., 2012; Duncan et al., 2015
	↑	Mouse	Harris et al., 2015
	↑	Human	Harris et al., 2015
SIRT1	↑	Mice	Kim et al., 2016
R1	↓	Human	Johnson et al., 2011

*5-HT, 5-hydroxytriptamine; CRH, corticotropin releasing hormone; CRH-R1, CRH receptor 1; KLF11, kruppel like factor 11; MAO-A, monoamine oxidase A; SERT, serotonin transporter; ↑, increase; ↓, decrease*.

### Glucocorticoids and social stress

Glucocorticoids are steroid hormones released from the adrenal glands in response to increased blood ACTH (Nardocci et al., 2014; Fink, 2017). Cortisol (hydrocortisone) is the main glucocorticoid in human, while corticosterone is the main glucocorticoid in rodents (Spackman and Riley, 1978; MacFarsane, 1984). Functions of glucocorticoids are mediated by the glucocorticoid receptor, which is categorized in the superfamily of nuclear receptors. The glucocorticoid receptor comprises a DNA-binding domain including two zinc finger motifs in its C-terminal portion (Beato and Sánchez-Pacheco, 1996). In the absence of glucocorticoid, glucocorticoid receptor forms a multiprotein complex with heat shock proteins in the cytosol. However, binding of glucocorticoid to glucocorticoid receptor induces the dissociation of the multiprotein complex in the cytosol and allow glucocorticoid receptor to translocate into the nucleus, in which glucocorticoid receptor may function as a transcriptional regulator of target genes (Reviewed in Grad and Picard, 2007; Lanfumey et al., 2008; Oakley and Cidlowski, 2013). In a social stress model of mice, susceptible mice show higher cytosolic glucocorticoid receptor levels in the hippocampus than control and resilient mice, while resilient mice show higher nuclear glucocorticoid receptor levels in the hippocampus, implying that social stress induces nuclear translocation of hippocampal glucocorticoid receptor of resilient mice at higher rate than susceptible mice (Han et al., 2017).

Hypersecretion of glucocorticoids is associated with depressive symptoms in humans and depression-like behaviors in animal models (Sachar et al., 1970; Lee et al., 2002; Krishnan and Nestler, 2011). Sachar et al. (1970) reported the association between the severity of the depressive symptoms and plasma cortisol levels in patients with major depression. The increase in plasma glucocorticoids has been observed in rodent models exposed to social defeat stress (Berton et al., 1998; Keeney et al., 2006; Razzoli et al., 2007; Grunewald et al., 2012; Iñiguez et al., 2014).

Plasma glucocorticoid levels are often measured as an endogenous stress marker. However, only a few studies have addressed the direct effects of glucocorticoids on the 5-HT system. Corticosterone treatment decreases 5-HT_1A_ receptor in the hippocampus but increases the 5-HT_2_ receptor in the frontal cortex (Takao et al., 1997). Also, chronic corticosterone treatment induces the reduction in 5-HT_1A_ autoreceptor sensitivity in the dorsal raphe nucleus (Fairchild et al., 2003). These results suggest that the glucocorticoid released as a result of activation of HPA axis may change the function of 5-HT neurons during stress.

Glucocorticoids are associated with the regulation of brain MAO-A, which plays a major role in the biological response to stress. Human MAO-A promoter region has three consensus glucocorticoid response elements and a core promoter, which contains four SP1/R1 binding sites (Ou et al., 2006a). Dexamethasone, a synthetic glucocorticoid, activates the MAO-A gene expression by direct interaction of glucocorticoid receptor with one of the three glucocorticoid response elements and indirect interaction with SP1 or R1 transcriptional factors (Ou et al., 2006a). Glucocorticoid receptor is also involved in stress response in the 5-HT system. Maternal separation in rodents increase the hippocampal 5-HT turnover and glucocorticoid receptor expression; however, the effect of maternal separation on glucocorticoid receptor expression was fully blocked by ketanserin, a 5-HT_2/7_ receptor antagonist (Mitchell et al., 1990; Smythe et al., 1994). Furthermore, it is suggested by Laplante et al. (2002) that the regulation of hippocampal glucocorticoid receptor by 5-HT is mediated by 5-HT_7_ receptor.

## Regulation of MAO-A during social stress

**Figure 1:**
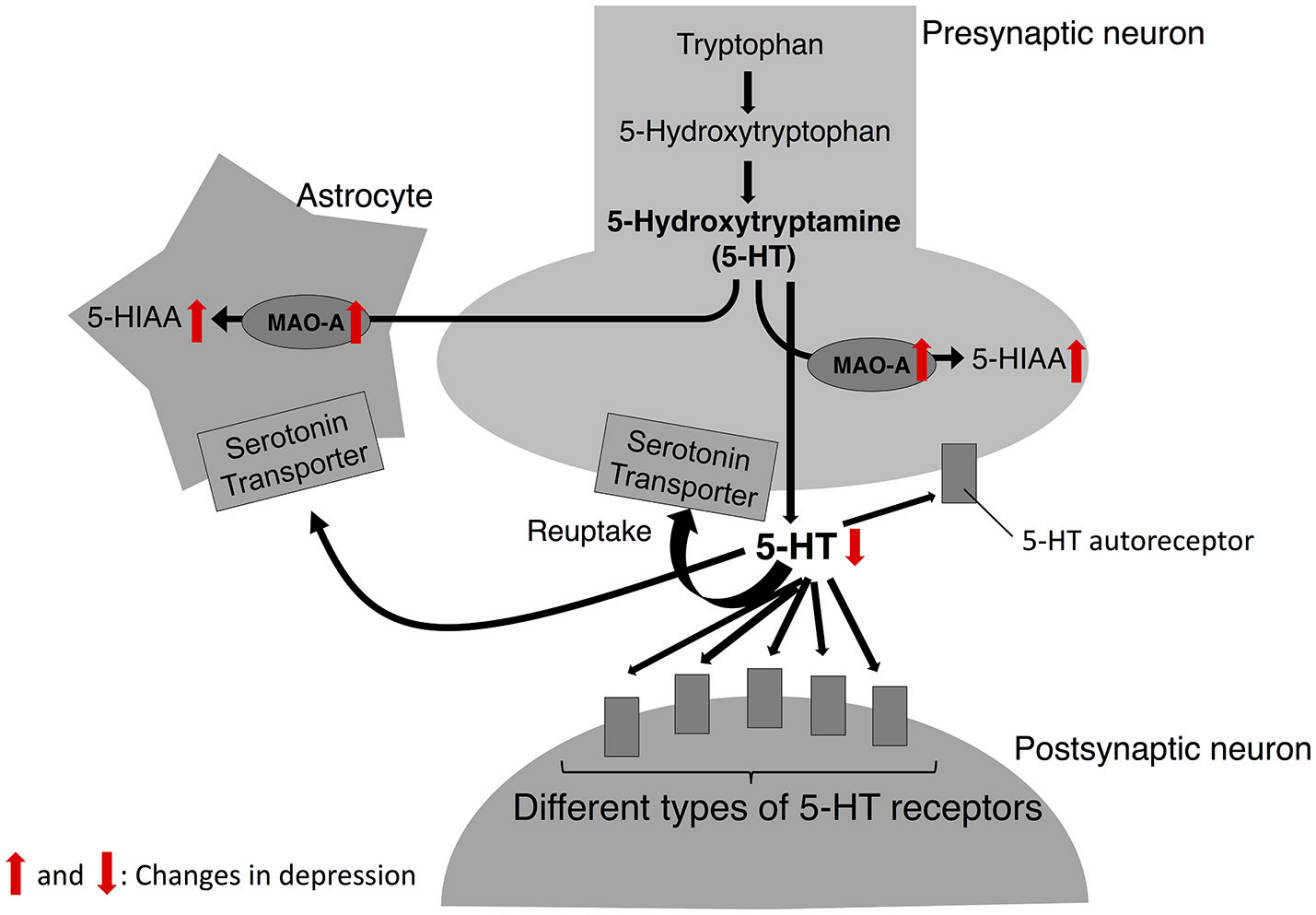
5-HT biosynthesis, release, and degradation. In serotonin theory of depression, the catalytic activity of MAO-A is upregulated in depression; thus, the levels of 5-HT released from 5-HT neurons are decreased while the levels of its metabolite, 5-HIAA are increased. Red arrows indicate the changes observed in depression.

The neurobiological mechanism underlying MDD has been studied for decades; however, it has not been fully elucidated. Researchers have suggested several theories explaining the neurobiological mechanism of depressive symptoms. One of the well-documented theories to account for the pathophysiology of depression is the serotonin theory (also known as serotonin hypothesis, monoamine theory, or monoamine hypothesis of depression, Figure 1). The development of this hypothesis dates to the 1950s when the effect of Iproniazid, an antitubercular drug, on the behavior of patients was reported (Robitzek et al., 1952). After this clinical discovery, researchers found that Iproniazid is capable of inhibiting MAO (Zeller et al., 1952) and increasing the 5-hydroxytriptamine (5-HT; serotonin) concentrations in the brain (Udenfriend et al., 1957). Based on these findings of Iproniazid, it has been hypothesized that 5-HT is the most important monoamine in the pathophysiology of depression (Coppen, 1967). It was hypothesized that lower 5-HT levels in the brain cause depressive symptoms. The first step of the 5-HT synthesis is the conversion of tryptophan to 5-Hydroxytryptophan (5-HTP). In the second phase, 5-HTP is decarboxylated to 5-HT by 5-HTP decarboxylase. The 5-HT is then packed into vesicles, released into the synaptic cleft, which then acts on different types of 5-HT receptors in pre- and post-synaptic neurons. Also, 5-HT is transported from the synaptic cleft to the pre-synaptic neuron by serotonin transporter (SERT) and recycled for future release. Degradation of 5-HT to 5-hydroxyindoleacetic acid (5-HIAA) is mediated mainly by MAO-A (Reviewed in aan het Rot et al., 2009). Social stress increases the 5-HIAA/5-HT ratios, an index of serotonin turnover (Blanchard et al., 1991; Winberg and Lepage, 1998; Overli et al., 1999; Beitia et al., 2005). In a recent positron emission tomography of human depression, it has been revealed that several neuronal factors in the 5-HT system are affected in the depressed patients (Reviewed in Smith and Jakobsen, 2013). For example, SERT in some brain regions is decreased in depressed patients (Miller et al., 2009b; Reimold et al., 2011; Kambeitz and Howes, 2015). As for 5-HT receptors, studies have reported changes in 5-HT_1A_ receptor levels (López-Figueroa et al., 2004; Miller et al., 2009a; Parsey et al., 2010) and 5-HT_1B_ receptor (Belzeaux et al., 2010; Murrough et al., 2011) in patients with depression. In animal models of social stress, chronic social stress decreases SERT and 5-HT_1A_ receptor mRNA levels (Boyarskikh et al., 2013). Findings about influence of social stress and depression on 5-HT system are summarized in Table 1.

Researchers have focused on the development of drugs that increase the 5-HT levels in the synaptic clefts, mainly by targeting SERT and several types of 5-HT receptors. In the brain, 5-HT is degraded primarily by MAO-A. Inhibitors of MAO-A have been used as antidepressants in the past; however, after the introduction of selective serotonin reuptake inhibitor and serotonin-noradrenaline reuptake inhibitor, they are less commonly used in the therapeutics for depression due to their peripheral side effects. Nevertheless, MAO-A is often used as a biological marker in human brain imaging studies of psychiatric diseases, including depression (Meyer et al., 2009; Smith and Jakobsen, 2013; Kolla et al., 2016). Meyer and co-workers found that MAO-A densities in several brain regions (e.g., prefrontal cortex, hippocampus, and midbrain) are higher in patients with depression even in the recovery phase (Meyer et al., 2006, 2009). Also, they reported the association between the MAO-A densities in the anterior cingulate cortex and the prefrontal cortex and the recurrence of depressive symptoms (Meyer et al., 2009).

Metabolism of 5-HT is largely mediated by MAO-A; however, serotonergic neurons mainly contain MAO-B, which has a less enzymatic activity for 5-HT degradation than MAO-A (O'Carroll et al., 1983). High amount of MAO-A has been reported outside 5-HT neurons; in human glial cells (Westlund et al., 1988), which is debatable (Richards et al., 1992), and rat astrocytes (Fitzgerald et al., 1990). Although, SERT is distributed exclusively in 5-HT neurons (Fujita et al., 1993), several lines of *in vitro* evidence suggest that SERT may mediate uptake of 5-HT into astrocytes. Studies using rodent primary astrocytes in culture show 5-HT uptake by SERT into the astrocytes (Bel et al., 1997; Hirst et al., 1998; Malynn et al., 2013). In addition, human astrocytes in culture express mRNA of SERT (Kubota et al., 2001) and 5-HT oxidization to 5-HIAA by MAO-A has been observed in rat primary astrocytes in culture (Fitzgerald et al., 1990). Therefore, astrocytes might contribute to the metabolism of 5-HT release from 5-HT neurons. However, the presence of SERT in astrocytes is controversial (Blakely et al., 1994; Bengel et al., 1997). Furthermore, the uptake of 5-HT can also be mediated by organic cation transporter 3, which is expressed not only in astrocytes but also in neurons (Takeda et al., 2002; Vialou et al., 2008; Gasser et al., 2009). In addition, plasma membrane monoamine transporter (PMAT), which transports many types of neurotransmitters, including 5-HT and dopamine (Engel et al., 2004; Engel and Wang, 2005), is expressed broadly in the brain (Dahlin et al., 2007). PMAT is mainly co-expressed with SERT in the neurons, rather than astrocytes (Dahlin et al., 2007). However, expression of PMAT is also observed in the brain regions where SERT is not expressed significantly, such as the cerebellum, accumbens shell, dentate gyrus of the hippocampus, and several parts in the forebrain cortex, indicating that PMAT is highly involved in 5-HT clearance in these regions (Dahlin et al., 2007). Therefore, it is likely that 5-HT released into the synaptic cleft can be taken up and metabolized by non-5-HT neurons.

MAO-A is also present in pyramidal cells in the orbitofrontal cortex and basolateral amygdala, and its role in proliferation and remodeling of apical dendrites has been shown (Godar et al., 2015). Thus, the metabolism of 5-HT outside of 5-HT nerve terminals, such as in astrocytes and in pyramid cells, can be significantly important.

MAO-A inhibitors are not the first choice in current medication for depressive disorders due to their adverse effects caused by their interaction with food and other drugs. In the presence of MAO-A inhibitors, food tyramine is not metabolized by MAO-A and absorbed excessively into the blood, causing hypertension (Horwitz et al., 1964; Anderson et al., 1993). MAO-A inhibitors can also cause severe adverse effects, such as disruption in thermoregulation and hypertensive crisis (so-called serotonin syndrome) when they are administered simultaneously with other drugs that increase 5-HT (Sun-Edelstein et al., 2008). Inhibition of MAO-A density by MAO-A inhibitor has been observed in many brain regions, including the anterior cingulate cortex, the prefrontal cortex, and temporal cortex, putamen, thalamus, hippocampus, and midbrain (Sacher et al., 2011). Furthermore, MAO-A inhibitors distributed in peripheral organs may act on MAO-A in these organs (Fowler et al., 2003). Inhibition of MAO-A in non-specific sites, including peripheral tissues and brain regions which are less likely to be involved in the pathology of depression, might cause severe adverse effects. Therefore, understanding the regulatory pathways of MAO-A in the brain and detecting brain regions critical to the function of MAO-A in the neuropathology of depression might give us insights into the development of new therapeutic strategy. In addition, to identify novel drug targets for 5-HT metabolism in specific brain regions will be a better option with less or no adverse effects as conventional MAO-A inhibitors.

### KLF11-MAO-A pathway in social stress

**Figure 2:**
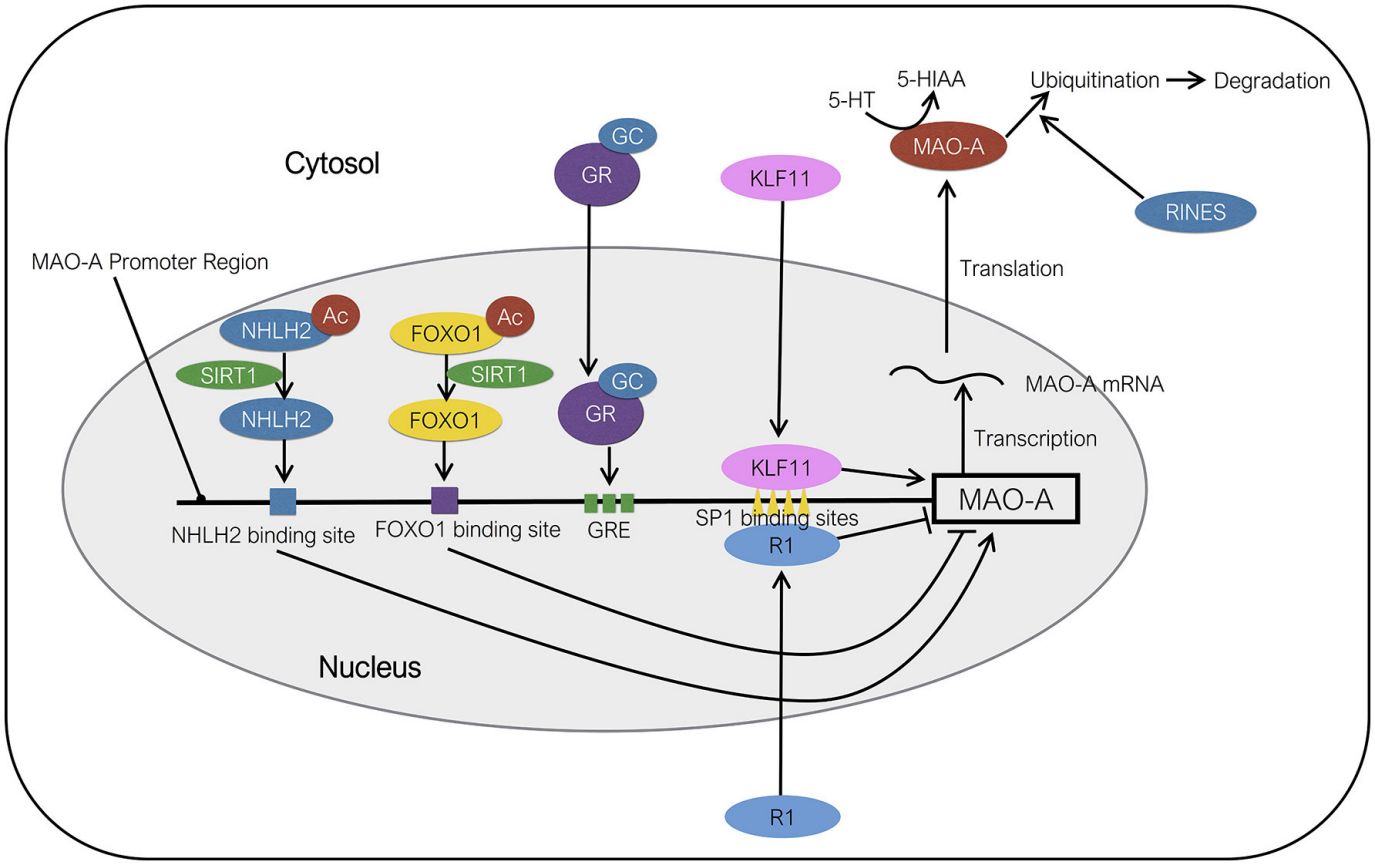
Molecular regulation of MAO-A expression. KLF11 activates the transcription of MAO-A by binding to SP1 binding sites. NHLH2 deacetylated by SIRT1 acts as a transcriptional activator of MAO-A. FOXO1 deacetylated by SIRT1 functions as a transcriptional suppressor of MAO-A. RINES promotes the ubiquitination and degradation of MAO-A protein. R1 represses the transcription of MAO-A gene by binding to SP1 binding sites. The order of protein binding sites in the MAO-A promoter region is illustrated ambiguously. Ac, acetyl group; GC, glucocorticoids; GR, glucocorticoid receptor; GRE, GC response element.

Kruppel like factor 11 (KLF11), also referred to as transforming growth factor-β-inducible early gene 2 (TIEG2), is a member of the KLF family of proteins. KLF11 contains three SP1-like Zn finger arrays at C-terminus and has a high degree of homology with KLF10 (Cook et al., 1998). KLF11 immunoreactivity was observed in the amygdala, frontal cortex, medial prefrontal cortex, dentate gyrus, CA2/3, CA1, hypothalamus, and thalamus (Duncan et al., 2015). KLF11 protein can regulate the expression of various genes by binding to GC-rich consensus SP1-like binding sites of promoter regions of the genes (Figure 2). Core promoter of MAO-A contains four SP1/KLF binding sites in addition to three glucocorticoid response elements (Ou et al., 2006a). It has been suggested that KLF11 can mediate glucocorticoid-induced up-regulation of MAO-A mRNA, protein, enzymatic activities owing to the fact that (1) glucocorticoids increase KLF11 mRNA and protein levels, (2) KLF11 overexpression increases MAO-A gene expression levels and enzymatic activity, which is further promoted by glucocorticoids, while KLF11 knockdown mediated by si-RNA decrease the MAO-A gene expression and enzymatic activity (Grunewald et al., 2012). It is also reported that glucocorticoids induce the translocation of the KLF11 protein from cytosol to the nucleus (Grunewald et al., 2012), although the molecular mechanism of the translocation remains unknown.

There are several studies demonstrating the association between KLF11 and social stress. Exposure to chronic social defeat stress increases KLF11-immunoreactivity in the frontal cortex, medial prefrontal cortex and CA1 in the hippocampus of rats (Duncan et al., 2015) where reduction in hippocampal volume has been found in imaging studies of MDD patients (Bremner et al., 2000; Bremner, 2002; Koolschijn et al., 2009; Cobb et al., 2013). Recently, Harris et al. (2015) found higher KLF11 and MAO-A protein levels in the prefrontal cortex of postmortem patients with depression, implying that KLF11 is involved in the up-regulation of MAO-A observed in patients with MDD. These findings of upregulation of KLF11 in the frontal cortex and prefrontal cortex indicates the possibility that KLF11 can be a prominent drug target to modulate MAO-A activity in certain brain regions related to depression. However, the influence of upregulation of MAO-A in these specific brain regions on 5-HT levels and postsynaptic signaling remains to be elucidated. It would also be necessary to evaluate the effects of 5-HT metabolism in these brain regions on depressive behavior in humans and animal models.

### SIRT1-MAO-A pathway

Silent mating type information regulation 2 homolog 1 (SIRT1) is a protein in the Sirtuin family. In mammals, there are seven Sirtuins, SIRT1-7. All of them possess the NAD^+^-binding site and common catalytic domain. However, they differ in their N- and C- terminal structures (Frye, 2000), substrates, protein binding partners and sub-cellular localization (Reviewed in Guarente, 2011; Donmez and Outeiro, 2013). Among the seven types of SIRT family of proteins, an association between SIRT1 and depression has been found in several studies (Kishi et al., 2010; Abe et al., 2011; Libert et al., 2011; Cai et al., 2015).

SIRT1 protein is distributed throughout the brain of rodents. With respect to the sub-cellular localization in the brain, SIRT1 is predominantly localized in the nucleus in the prefrontal cortex, hippocampus, substantia nigra, and spinal cord regions (Zakhary et al., 2010). However, sub-cellular localization varies in different types of cells. SIRT1 protein is expressed mainly in the cytoplasm of the neurons in the striatum, while it is found both in nucleus and cytoplasm of the ependymal cells (Tanno et al., 2007).

SIRT1 is an NAD^+^-dependent protein deacetylase, which can deacetylate some transcriptional factors associated with neuronal protection, resistance to oxidative stress, fatty acid oxidation, glucose production, and mitochondrial biogenesis (Reviewed in Donmez and Outeiro, 2013). An association between SIRT1 single nucleotide polymorphism and several psychiatric disorders, including social phobia (rs12778366), anxiety disorder (rs10997870), and panic disorder (rs12778366 and rs10997870) has been reported which indicates that SIRT1 is associated with mental disorders in humans (Libert et al., 2011). An association between SIRT1 SNP (rs10997875) and MDD in Japanese subjects has also been reported (Kishi et al., 2010). Furthermore, patients with MDD show significantly lower SIRT1 mRNA levels in peripheral white blood cells in depressive states whereas this reduction in SIRT1 mRNA is not observed in MDD patients in remissive state, implying that SIRT1 in peripheral white blood cells could be utilized as a potential state-dependent biomarker for MDD (Abe et al., 2011). However, relationship between SIRT1 in peripheral tissues and the brain remains unknown. Therefore, we need to further investigate how brain SIRT1 contributes to the manifestation of depression and how it correlates with the peripheral SIRT1 levels to assess the clinical utility of SIRT1 as a biological marker for depression.

In animal models, chronic social defeat stress increases SIRT1 mRNA and protein levels in the nucleus accumbens (Kim et al., 2016). Brain-specific SIRT1 knockout mice show lower MAO-A gene expression levels, higher 5-HT levels, lower 5-HIAA levels in the brain and less susceptibility to social defeat stress, compared to wild-type mice (Libert et al., 2011). On the other hand, SIRT1 overexpression mice show higher MAO-A gene expression levels, lower 5-HT levels, and higher 5-HIAA levels in the brain, compared to wild-type mice (Libert et al., 2011). These results indicate the role of SIRT1 in the regulation of MAO-A, which can alter the 5-HT metabolism in the brain.

The molecular mechanism underlying the action of SIRT1 in the 5-HT system remains to be elucidated. So far two regulatory factors, the brain specific nescient helix-loop-helix transcription factor [NHLH2; also known as neural basic helix-loop-helix transcription factor-2 (NSCL-2); Figure 2] and the Forkhead box O-1 (FOXO-1; Figure 2) have been identified as a substrate of SIRT1 and a potential medium between SIRT1 and MAO-A. NHLH2 deacetylated on lysine 49 by SIRT1 can activate the transcription of MAO-A by binding to NHLH2-binding sites in the MAO-A promoter region (Libert et al., 2011). FOXO1 is involved in the valproate-induced activation of MAO-A gene expression, catalytic activity, and promoter activity. FOXO1 represses the transcription of MAO-A by directly binding to a FOXO1-binding site of the MAO-A promoter (Wu and Shih, 2011). An *in vitro* study revealed that SIRT1 deacetylates the FOXO1 and represses its activity in HeLa cells (Motta et al., 2004). These results indicate that NHLH2 acts as an activator of 5-HT metabolism, while FOXO1 acts as a repressor in SIRT1 signaling. However, precise roles of the NHLH2 and FOXO1 in the regulation of central 5-HT system under social stress have not been fully elucidated.

SIRT1 is an interesting protein which can regulate the activities of the transcriptional activator (i.e., NHLH2) and repressor (i.e., FOXO1) of MAO-A. Effects of the SIRT1 on NHLH2 and FOXO1 have been observed *in vitro* studies (Libert et al., 2011; Wu and Shih, 2011); however, the brain region-specific effects of SIRT1 on these transcriptional factors under stress are yet to be explained. Therefore, it might be important to know the brain regions where the SIRT1-NHLH2-MAO-A or SIRT1-FOXO1-MAO-A pathways are playing important roles in the physiological and behavioral changes caused by social stress.

### RINES (ring finger nuclease 180) in the degradation of MAO-A

RINES (An abbreviation of ring finger protein in neural stem cells), also known as ring finger nuclease 180 (RNF180), is a protein which contains protein binding sites, a basic coiled-coil domain, and a ring finger domain (Ogawa et al., 2008). RINES protein has been detected in the olfactory bulb, cerebellum, thalamus, striatum, cerebral cortex, midbrain, pons, amygdala, and hippocampus in mice. The expression of RINES mRNA has been reported in CA3, dentate gyrus of hippocampus; prefrontal cortex, amygdala, nucleus accumbens, corpus striatum, locus coeruleus, substantia nigra, and raphe nuclei in mice (Kabayama et al., 2013). RINES knockout mice show enhanced anxiety behavior, abnormal stress response (passive avoidance test and forced swimming test) and increased social interaction in resident-intruder paradigm. RINES knockout mice also show significantly higher MAO-A catalytic activity specifically in the locus coeruleus, but not in the other regions, including the basolateral nucleus of the amygdala, prefrontal cortex, raphe nuclei and substantia nigra, than the wild-type mice (Kabayama et al., 2013). In the same study, the authors found that RINES promotes the ubiquitination and degradation of MAO-A protein *in vitro*. These results imply that RINES is a potential modulator of central MAO-A in a specific brain region (Kabayama et al., 2013; Figure 2). However, the role of brain RINES under social stress and interaction with other factors, such as HPA-axis, has not been reported.

Among the proteins introduced in this review, RINES is the only factor which is known to be involved in the degradation of MAO-A, although the other proteins act as transcriptional factors in the regulation of MAO-A. Regulation of degradation of MAO-A under stress has not been well studied.

### R1 in MAO-A regulation

R1 [also known as Cell division cycle-associated 7-like protein (CDCA7L)] represses the transcription of MAO-A gene by binding to SP1-binding sites of MAO-A promoter (Figure 2). R1 protein contains several important functional regions, including Pro, Glu, Ser, Thr-rich region, nuclear targeting region, and DNA binding region. Pro, Glu, Ser, Thr-rich region is known to be susceptible to degradation by the proteasome. The existence of the nuclear binding region suggests that R1 has an ability to translocate between nucleus and cytosol. In addition, DNA binding region containing four zinc finger domains have been identified, suggesting that R1 is a transcription factor (Chen et al., 2005). Levels of R1 protein in the prefrontal cortex of postmortem patients diagnosed with MDD are significantly lower than those of healthy control subjects (Johnson et al., 2011). R1 also represses the transcription of MAO-A gene, implying the involvement of R1 in the 5-HT system (Chen et al., 2005; Ou et al., 2006a,b). Furthermore, the translocation of R1 from cytosol to nucleus is promoted by dexamethasone, a synthetic glucocorticoid, *in vitro* (Ou et al., 2006a). However, distribution of R1 protein in the brain and influence of social defeat stress on R1 in MAO-A regulatory system have not been elucidated. Therefore, detecting the brain regions where social defeat stress affects the expression of R1, MAO-A, and its substrates, including 5-HT, would give us an important key to understand the role of R1 in the regulation of MAO-A under stress.

## Conclusion

There is evidence that social stress can lead to depression, which is mediated by the HPA-axis and the central 5-HT system. MAO-A inhibitors are selectively used in therapeutics for depression due to their adverse effects (Shulman et al., 2009; Menkes et al., 2016) caused by the inhibition of MAO-A in non-specific brain regions. MAO-A is an essential enzyme for normal brain function. Therefore, it is important to maintain endogenous MAO-A activity within the normal range is important to avoid unwanted side effects caused by direct inhibition of MAO-A by exogeneous inhibitors. In fact, human imaging studies has shown that MAO-A density in the brain after MAO-A inhibitor treatment is lower than that of psychiatrically normal subjects (Sacher et al., 2011). Modulation of endogenous levels of the MAO-A regulatory factors could be an alternative approach to keep normal MAO-A levels in the brain. In addition, contribution of the regulatory factors to MAO-A levels can be different depending on the brain regions. Malfunction of RINES in RINES knockout mice shows an increase of MAO-A activity only in the locus coeruleus, although RINES mRNA and protein are distributed in many other brain regions in wild-type animals. This result indicates that RINES has significant contribution to the regulation of MAO-A expression and protein levels only in this specific brain region (Kabayama et al., 2013). Therefore, contribution of the regulatory factors to MAO-A levels can vary among brain regions, which might enable us to indirectly target MAO-A in specific brain regions by modulating the regulatory factors. Furthermore, MAO-A inhibitors are more effective for only some subtypes of depression (McGrath et al., 1993; Søgaard et al., 1999; Henkel et al., 2006; Shulman et al., 2013). Therefore, it is important to elucidate the molecular pathways that regulate MAO-A in the brain to develop novel drug targets for the control of MAO-A activity. KLF11, R1 and SIRT1 modulate 5-HT neuronal morphology (Ou et al., 2006b; Duncan et al., 2015; Abe-Higuchi et al., 2016), levels of MAO-A and 5-HT in the brain. Colocalization of these regulatory factors with MAO-A, and the identification of their sites of action (brain regions and cell types), along with their stress-related change would give an insight into the development of depression. These MAO-A regulatory factors could then be potential drug targets for depression. Since depression can be categorized into two subtypes: hypercortisolemic and non-hypercortisolemic (Carroll et al., 2007); therefore, further research is necessary to identify if the MAO-A regulatory mechanisms operate differently under different HPA conditions. Information from high resolution imaging studies of MAO-A in the human brain, in parallel with the development of depression, might be important to develop drugs that target specific brain regions as therapeutics for depression.

## Author contributions

YH wrote this review paper. IP and TS edited.

### Conflict of interest statement

The authors declare that the research was conducted in the absence of any commercial or financial relationships that could be construed as a potential conflict of interest.
